# Supplementary material for: Differences between atrial fibrillation diagnosed before and after stroke: A large real-world cohort study
Source: PLoS One. 2024 Aug 14;19(8):e0308507. doi: 10.1371/journal.pone.0308507 (PMC11324098; doi:10.1371/journal.pone.0308507)
Supplement: S1 Table — (DOCX) [file pone.0308507.s001.docx]

| **S1 Table. ICD-9-CM and ICD-10-CM codes for diagnosis of risk factors and comorbidities** | |
| --- | --- |
| Comorbidities | ICD-9-CM and ICD-10-CM codes for diagnosis |
| Hypertension | ICD-9-CM codes: 401.0–401.9, 402.00–402.91, 403.00–403.91, 404.00–404.93, 405.01–405.99, 437.2, 348.2, 997.91; ICD-10-CM codes: G93.2, I10, I27.0 |
| Diabetes mellitus | ICD-9-CM: 250.00–250.01, 250.20, 250.21, 250.80, 250.81, 250.82, 250.83; ICD-10-CM: E8.00, E8.01, E09.00, E09.11, E10.610, E10.618, E10.620-622, E10.628, E10.630, E10.638, E10.65, E10.69, E10.9, E11.00, E11.01, E11.610, E11.618, E11.620-622, E11.628, E11.630, E11.638, E11.649, E11.65, E11.69, E11.9, E13.00, E13.01, E13.9 |
| Hyperlipidemia | ICD-9-CM: 272.0-272.4; ICD-10-CM: E78.0-E78.5 |
| Coronary artery disease | ICD-9-CM: 414.00-414.07, 414.2, 414.3, 414.4; ICD-10: I25.1 |
| Heart failure | ICD-9-CM: code 428; ICD-10-CM: I50 |
| Peripheral vascular disease | ICD-9-CM: 440.20, 440.21, 440.23, 440.24, 440.9, 443.81, 443.9, 444.22; ICD-10-CM: I70.211-219, I70.25, E70.231-249, I70.90, I70.92, I79.1, I79.8 |
| Chronic kidney disease | ICD-9-CM: 585; ICD-10-CM: N18.1-N18.6, N18.9 |
| Chronic obstructive pulmonary disease | ICD-9-CM: 490-496; ICD-10-CM: J40, J41.0, J41.1, J41.8, J42, J43.9, J44.0, J44.1, J44.9, J45.20-J45.22, J45.901, J45.902, J45.909, J45.990, J45.991, J45.998, J47.1, J47.9, J67.0-J67.9 |
| Prior stroke/TIA | ICD-9-CM: 430-434, V12.459; ICD-10-CM: I60-I63, I65, I66, Z86.73 |
| Ischemic stroke | ICD-9-CM=433, 434, ICD-10-CM= I63, I65, I66 |
| Hemorrhagic stroke | ICD-9-CM=430-432, ICD-10-CM=I60-I62 |

ICD: international classification of diseases; CM: clinical modification; TIA: transient ischemic attack
