# Supplementary material for: Differences between atrial fibrillation diagnosed before and after stroke: A large real-world cohort study
Source: PLoS One. 2024 Aug 14;19(8):e0308507. doi: 10.1371/journal.pone.0308507 (PMC11324098; doi:10.1371/journal.pone.0308507)
Supplement: S2 Table — (DOCX) [file pone.0308507.s002.docx]

| **S2 Table. Multivariable Cox regression models to predict the outcome of recurrent ischemic stroke and hemorrhage stroke at the end of follow-up** | | | |
| --- | --- | --- | --- |
| Outcome Measures | Non-AF | AFADS | KAF |
| Recurrent ischemic stroke | | | |
| n(%) | 24341(18.1) | 1763(22.5) | 3503(21.0) |
| HR (95%CI)# | Ref. | 1.69(1.61-1.78)** | 1.47(1.41-1.54)** |
| HR (95%CI)# | 0.59(0.56-0.62)** | Ref. | 0.87(0.82-0.92)** |
| Hemorrhage stroke | | | |
| n(%) | 6517(4.8) | 503(6.4) | 890(5.3) |
| HR (95%CI)# | Ref. | 1.71(1.58-1.85)** | 1.56(1.46-1.67)** |
| HR (95%CI)# | 0.59(0.54-0.63)** | Ref. | 0.91(0.84-0.99)* |

KAF: known atrial fibrillation; AFDAS: atrial fibrillation detected after stroke; Non-AF: without atrial fibrillation; CI: confidence interval; Ref.: reference.

#Adjusted age, sex, stroke severity index score, comorbidities (hypertension, diabetes, hyperlipidemia, coronary artery disease, heart failure, peripheral artery disease, chronic kidney disease, prior stroke/TIA), modified Charlson Comorbidity Index score, anticoagulant treatment and death.

*P<0.01; **P<0.001.
