# Supplementary material for: Differences between atrial fibrillation diagnosed before and after stroke: A large real-world cohort study
Source: PLoS One. 2024 Aug 14;19(8):e0308507. doi: 10.1371/journal.pone.0308507 (PMC11324098; doi:10.1371/journal.pone.0308507)
Supplement: S3 Table — (DOCX) [file pone.0308507.s003.docx]

**S3 Table. Multivariable Cox regression models to predict the outcome of recurrent ischemic stroke, hemorrhage stroke, or death at the end of follow-up in AF patients with and without OAC therapy.**

| Outcome Measures | No OAC | OAC |
| --- | --- | --- |
| Recurrent ischemic stroke |  |  |
| n(%) | 3341(31.51) | 1815(12.00) |
| HR (95%CI)* | Ref. | 0.20(0.19-0.21)** |
| Hemorrhage stroke |  |  |
| n(%) | 1346(14.36) | 889(5.44) |
| HR (95%CI)* | Ref | 0.20(0.18-0.22)** |
| Death |  |  |
| n(%) | 5404(63.49) | 3842(22.32) |
| HR (95%CI)* | Ref. | 0.26(0.25-0.27)** |

AF: atrial fibrillation; OAC: oral anticoagulant; CI: confidence interval; Ref.: reference.

#Adjusted age, sex, stroke severity index score, comorbidities (hypertension, diabetes, hyperlipidemia, coronary artery disease, heart failure, peripheral artery disease, chronic kidney disease, prior stroke/TIA), modified Charlson Comorbidity Index score.

*P<0.01; **P<0.001.
