# Supplementary material for: Differences between atrial fibrillation diagnosed before and after stroke: A large real-world cohort study
Source: PLoS One. 2024 Aug 14;19(8):e0308507. doi: 10.1371/journal.pone.0308507 (PMC11324098; doi:10.1371/journal.pone.0308507)
Supplement: S4 Table — (DOCX) [file pone.0308507.s004.docx]

**S4 Table. Multivariable Cox regression models to predict the outcome of recurrent ischemic stroke, hemorrhage stroke, or death at the end of follow-up in KAF patients with and without OAC therapy.**

| Outcome Measures | No OAC | OAC |
| --- | --- | --- |
| Recurrent ischemic stroke |  |  |
| n(%) | 1921(28.15) | 1099(12.73) |
| HR (95%CI)* | Ref. | 0.24(0.22-0.25)** |
| Hemorrhage stroke |  |  |
| n(%) | 754(12.24) | 533(5.73) |
| HR (95%CI)* | Ref | 0.24(0.22-0.27)** |
| Death |  |  |
| n(%) | 3666(64.36) | 2406(24.64) |
| HR (95%CI)* | Ref. | 0.27(0.26-0.29)** |

KAF: known atrial fibrillation; OAC: oral anticoagulant; CI: confidence interval; Ref.: reference.

#Adjusted age, sex, stroke severity index score, comorbidities (hypertension, diabetes, hyperlipidemia, coronary artery disease, heart failure, peripheral artery disease, chronic kidney disease, prior stroke/TIA), and modified Charlson Comorbidity Index score.

*P<0.01; **P<0.001.
