# Supplementary material for: Differences between atrial fibrillation diagnosed before and after stroke: A large real-world cohort study
Source: PLoS One. 2024 Aug 14;19(8):e0308507. doi: 10.1371/journal.pone.0308507 (PMC11324098; doi:10.1371/journal.pone.0308507)
Supplement: Attachment — Submitted filename: Response to Reviewers V4.docx [file pone.0308507.s006.docx]

**Response to Reviewers’ comments**

**Ref.: PONE-D-23-43796R1**

Reviewers’ comments:

Must include:

- A rebuttal letter that responds to each point raised by the academic editor and reviewer(s). You should upload this letter as a separate file labeled 'Response to Reviewers'.
- A marked-up copy of your manuscript that highlights changes made to the original version. You should upload this as a separate file labeled 'Revised Manuscript with Track Changes'.
- An unmarked version of your revised paper without tracked changes. You should upload this as a separate file labeled 'Manuscript'.

**Reply**: Thank you. We had attached the files in including the rebuttal letter, marked-up and unmarked manuscripts, respectively.

Journal Requirements: When submitting your revision, we need you to address these additional requirements.

1. Please ensure that your manuscript meets PLOS ONE's style requirements, including those for file naming. The PLOS ONE style templates can be found at
   <https://journals.plos.org/plosone/s/file?id=wjVg/PLOSOne_formatting_sample_main_body.pdf> and 
   <https://journals.plos.org/plosone/s/file?id=ba62/PLOSOne_formatting_sample_title_authors_affiliations.pdf>

**Reply**: Thank you. Please see Manuscript_Clean file. We had revised the manuscript according to PLOS ONE's style.

2. We note that the grant information you provided in the ‘Funding Information’ and ‘Financial Disclosure’ sections do not match. When you resubmit, please ensure that you provide the correct grant numbers for the awards you received for your study in the ‘Funding Information’ section.

**Reply**: Thank you for your comment. We had added the ‘Funding Information’ section in the manuscript ((Please see Manuscript_Clean file P19L28-31)

3. Thank you for stating the following financial disclosure: This work was funded by grants from Tungs’ Taichung Metroharbor Hospital (protocol number TTMHH-109R0005). Please state what role the funders took in the study.  If the funders had no role, please state: "The funders had no role in study design, data collection and analysis, decision to publish, or preparation of the manuscript." If this statement is not correct you must amend it as needed. Please include this amended Role of Funder statement in your cover letter; we will change the online submission form on your behalf.

**Reply**: Thank you. The funders had no role in this study. We had added the statement "The funders had no role in study design, data collection and analysis, decision to publish, or preparation of the manuscript." in the financial disclosure section (Please see Manuscript_Clean file P19L29-31).

4. Thank you for stating the following in the Acknowledgments Section of your manuscript: This work was funded by grants from Tungs’ Taichung Metroharbor Hospital (protocol number TTMHH-109R0005). We note that you have provided funding information that is not currently declared in your Funding Statement. However, funding information should not appear in the Acknowledgments section or other areas of your manuscript. We will only publish funding information present in the Funding Statement section of the online submission form. Please remove any funding-related text from the manuscript and let us know how you would like to update your Funding Statement. Currently, your Funding Statement reads as follows: This work was funded by grants from Tungs’ Taichung Metroharbor Hospital (protocol number TTMHH-109R0005).Please include your amended statements within your cover letter; we will change the online submission form on your behalf.

**Reply**: Thank you for your suggestions. Please see Manuscript_Clean file. We had moved the sentence “This work was funded by grants from Tungs’ Taichung Metroharbor Hospital (protocol number TTMHH-109R0005).” to Funding Statement.

5. When completing the data availability statement of the submission form, you indicated that you will make your data available on acceptance. We strongly recommend all authors decide on a data sharing plan before acceptance, as the process can be lengthy and hold up publication timelines. Please note that, though access restrictions are acceptable now, your entire data will need to be made freely accessible if your manuscript is accepted for publication. This policy applies to all data except where public deposition would breach compliance with the protocol approved by your research ethics board. *If you are unable to adhere to our open data policy, please kindly revise your statement to explain your reasoning and we will seek the editor's input on an exemption*. Please be assured that, once you have provided your new statement, the assessment of your exemption will not hold up the peer review process.

**Reply**: Thank you for your comment. We had appended the below sentences “Data are available from the National Health Insurance Research Database (NHIRD) published by Taiwan National Health Insurance (NHI) Bureau. Due to legal restrictions imposed by the government of Taiwan in relation to the “Personal Information Protection Act”, data cannot be made publicly available. Requests for data can be sent as a formal proposal to the NHIRD (https://dep.mohw.gov.tw/DOS/cp-5119-59201-113.html).” in the ‘Data Availability Statement’. (Please see Manuscript_Clean file P20L12-18)

6. We notice that your supplementary tables are included in the manuscript file. Please remove them and upload them with the file type 'Supporting Information'. Please ensure that each Supporting Information file has a legend listed in the manuscript after the references list.

**Reply**: Thank you. We had upload the S1 Table as a separated file with the file type 'Supporting Information'.

**Reviewer #1:**

In this study, the authors retrospectively investigated more than 160.000 thousand patients from the National Health Insurance Research Database (NHIRD) of the NHI program in Taiwan. The aims of this study were to evaluate the clinical characteristics of newly diagnosed atrial fibrillation after a stroke (AFADS) in patients with ischemic stroke (IS) in a large population-based cohort; the differences in comorbidities and outcomes among the patients with known AF (KAF), AFADS, and without AF; the effects of anticoagulant treatment in patients with IS with either KAF or AFADS. The authors reported a very high mortality rate among patients with AF (both groups), and a higher risk of recurrent IS and hemorrhagic stroke for patients with KAF compared with AFADS.
The study is conducted in a large population and is overall well written, applying also a quite good statistical method. However, I have some major and minor comments:

**Major**
**1) It is not clear at which time the data regarding OAC therapy has been extracted from the registry. Is it after the stroke events? Please provide this data.**

**Reply:** Thank you for requesting this clarification. We apologize for not specifying the timing of the OAC prescription data extraction more clearly in our original submission. The data on OAC therapy for each patient was extracted from the National Health Insurance Research Database (NHIRD) based on prescriptions filled during the follow-up period after the index ischemic stroke hospitalization. Specifically, we captured all OAC prescriptions (warfarin or direct oral anticoagulants) for all patients from the time of their hospital discharge until the study end date, outcome event, or death.

We have now clarified this in the Methods section ((Please see Manuscript Clean file P5L17-21), stating: "Antithrombotic treatments of study subjects were ascertained from NHI drug codes and Anatomical Therapeutic Chemical (ATC) codes. Antithrombotic prescriptions (including antiplatelet agents, warfarin and DOACs) were extracted during the follow-up period after the index stroke hospitalization until the study end date, outcome event, or death." We appreciate you pointing out this ambiguity, as specifying the timing of exposure ascertainment is critical for transparency and interpretability of our findings.

**2) Following the previous point, many patients with KAF should have been already treated with OAC therapy before the stroke. I believe that it would be very interesting to know how many of them were already on treatment before the stroke.**

**Reply:** Thank you for raising this important point. To address this, we re-analyzed the data to determine the proportion of patients with known AF (KAF) who received oral anticoagulant (OAC) treatment prior to their index stroke. We found that 7304 (47.2%) patients with KAF had received OACs treatment within the 6 months preceding the index stroke. Adherence to OAC therapy was evaluated using the proportion of days covered (PDC), calculated by dividing the days a patient had medication by the days in the observation period. Patients with a PDC of ≥80% were considered adherent. Over the 6 months and 30 days prior to the index stroke, 4462 (26.7%) and 4068 (24.4%) patients, respectively, were adherent to OAC therapy.

We have added this new data to the Results (Please see Manuscript_Clean file P7L20-26) section: “In patients with KAF, 7304 (47.2%) patients had received OACs treatment within the 6 months preceding the index stroke. Adherence to OAC therapy was evaluated using the proportion of days covered (PDC), calculated by dividing the days a patient had medication by the days in the observation period. Patients with a PDC of ≥80% were considered adherent. Over the 6 months and 30 days prior to the index stroke, 4462 (26.7%) and 4068 (24.4%) patients, respectively, were adherent to OAC therapy.”

We also highlight the concerning issue of OAC undertreatment in patients with KAF In the Discussion section: “ Only about a quarter of our patients with KAF were adherent to OAC therapy before the occurrence of the index stroke. Although PDC provides an indirect measure of treatment adherence, our findings suggest that a substantial proportion of KAF patients were not receiving guideline-recommended OAC therapy before their stroke events. The low rates of OAC prescription and adherence in this population underscore the urgent need for improved OAC initiation and adherence in clinical practice to prevent disabling strokes in high-risk patients with KAF (Please see Manuscript_Clean file P14L21-31).

We appreciate you drawing attention to this crucial aspect of AF management. Thank you for this valuable suggestion.

**3) Since data were collected regarding antiplatelet therapy, direct oral anticoagulants (DOAC) and vitamin K antagonist (VKAs), it would be interesting to see the results of the cox regression analysis also for the subgroups of antithrombotic treatment, especially in regard of hemorragic events.**

**Reply:** Thank you for the comment. To investigate the impact of different antithrombotic therapies on the risks of outcome measures, we conducted multivariable Cox regression analyses comparing the hazard ratios (HRs) for hemorrhagic stroke associated with warfarin, direct oral anticoagulants (DOACs), antiplatelet therapy, and no antithrombotic treatment. The analyses were performed separately for all patients, those with known AF (KAF), those with AF diagnosed after stroke (AFADS), and those without AF (non-AF). In order to focus on hemorrhagic events, we summarized the results of hemorrhagic stroke in the Table listed below:

| Table. Summary of Multivariable Cox regression models to predict hemorrhage stroke among different antithrombotic treatments in different subgroups. | | | | |
| --- | --- | --- | --- | --- |
| Hemorrhagic stroke | Warfarin | DOAC | Antiplatelets | No Antithrombitics |
| All patients, n(%) | 116(8.62) | 487(4.80) | 8118(6.65) | 5435(17.75) |
| HR (95%CI)* | Ref. | 0.57(0.46-0.70)** | 0.81(0.67-0.97)* | 3.71(3.08-4.46)** |
| KAF, n (%) | 41(9.95) | 214(4.95) | 286(9.18) | 468(15.37) |
| HR (95%CI)* | Ref. | 0.49(0.35-0.69)** | 1.29(0.93-1.80) | 4.75(3.41-6.61)** |
| AFDAS, n(%) | 14(10.22) | 153(4.64) | 190(11.54) | 402(25.62) |
| HR (95%CI)* | Ref. | 0.47(0.27-0.81)* | 1.63(0.94-2.82) | 8.33(4.84-14.34)** |
| Non-AF, n(%) | 61(7.66) | 120(4.75) | 7642(6.51) | 4565(17.55) |
| HR (95%CI)* | Ref. | 0.67(0.49-0.91)* | 0.93(0.72-1.19) | 4.16(3.23-5.36)** |

KAF: known atrial fibrillation; AFDAS: atrial fibrillation detected after stroke; Non-AF: without atrial fibrillation; CI: confidence interval.

Ref.: reference.

#Adjusted age, sex, stroke severity index score, comorbidities (hypertension, diabetes, hyperlipidemia, coronary artery disease, heart failure, peripheral artery disease, chronic kidney disease, prior stroke/TIA), modified Charlson Comorbidity Index score.

*P<0.01; **P<0.001.

We present the main finding in the Results section: “DOACs were associated with significantly lower risks of hemorrhagic stroke compared to warfarin in KAF subgroup (HR 0.49, 95% CI: 0.35-0.69, p<0.001) and AFADS subgroup (HR 0.47, 95% CI: 0.27-0.81, p<0.001). The risks of hemorrhagic stroke were not significantly different between Warfarin and antiplatelet treatment in both KAF and AFDSA subgroups.” (Please see Manuscript_Clean file P8L23-27) to highlight the risks of hemorrhagic stroke related to different antithrombotic therapy for readers’ reference. We also add a brief statement in Discussion section: “ In addition, our results showed that DOACs have a superior safety profile with regard to hemorrhagic stroke risk compared to warfarin and antiplatelet therapy in patients with AF, whereas warfarin treatment was not associated with a higher risk of hemorrhagic stroke compared to antiplatelet treatment.” (Please see Manuscript_Clean file P12L15-19)

Thank you for raising the important point which help us to have deeper understandings about the benefits and risks of different antithrombotic treatments in our patients. Currently, we could not answer some questions such as the reasons for OACs use in patients without AF, effects and risks of OACs in patients without AF, the reasons for with-holding antithrombotic treatment in different subgroups, and the risk of withholding antithrombotic treatment in different patient subgroups. The results inspire us to explore more important (and complex) issues in the future study.

**4) Regarding the Cox regression analysis, the authors did not report about the adjustments made in the multivariable analysis for the risk of recurrent stroke, hemorragic stroke and all-cause mortality (Tables 2,3 and 4). Please provide this data.**

**Reply:** Thank you for noting this omission in our reporting of the multivariable Cox regression analyses. We apologize for not clearly specifying the variables adjusted for in the models presented in Tables 2, 3, and 4.

To clarify, the multivariable Cox regression models for the outcomes of recurrent ischemic stroke (Table 2), hemorrhagic stroke (Table 3), and all-cause mortality (Table 4) adjusted for the following covariates: age, sex, stroke severity index (SSI) score, Comorbidities (including hypertension, diabetes mellitus, hyperlipidemia, coronary artery disease, heart failure, peripheral artery disease, chronic kidney disease, prior stroke/TIA), Modified Charlson Comorbidity Index (CCI) score, and anticoagulant treatment. These covariates were selected a priori for adjustment based on their potential to confound the associations between AF status and stroke outcomes or mortality.

We have now clarified this in Methods section, under ‘Statistical Analysis’: “Multivariable Cox regression analyses were performed to estimate the HRs and 95% CIs for the association between AF subtype and the risk of recurrent ischemic stroke, hemorrhagic stroke, and all-cause mortality, adjusting for potential confounders including age, sex, stroke severity, comorbidities, and antithrombotic treatment.” (Please see Manuscript_Clean file P6L1-5), and added a footnote to each of Tables 2-4 stating: "Multivariable models adjusted for potential confounders including age, sex, stroke severity index score, comorbidities (hypertension, diabetes, hyperlipidemia, coronary artery disease, heart failure, peripheral artery disease, chronic kidney disease, prior stroke/TIA), modified Charlson Comorbidity Index score, and antithrombotic treatment."

Thank you for prompting us to clarify the rationale for covariate inclusion in our models. We believe these revisions will improve the interpretability of our approach. Please let us know if you have any additional questions or suggestions.

**5) Since the independent variable including the type of AF (eg. Non-AF, AFADS, KAF) was already studied in the different models for the various endpoints (results reported in table 2, 3 and 4), I do not understand why it has been done the same thing also in table 5. Furthermore, the results of the analysis using Non-AF as reference reported in table 5 are different compared to those in Tables 2,3 and 4, suggesting different adjustments. Please specify.**

**Reply:** Thank you for raising this important point about the potential overlap and differences in the analyses presented in Tables 2-4 versus Table 5. We apologize for any confusion caused by the presentation of the AF subtype variable across these tables. To clarify, in Tables 2-4, we included AF subtype (non-AF, AFADS, KAF) as an independent predictor in the multivariable Cox regression models for each outcome (recurrent ischemic stroke, hemorrhagic stroke, and all-cause mortality), along with other covariates for adjustment. The purpose of these analyses was to assess the independent associations of AF subtype with the outcomes, while controlling for potential confounders.

In Table 5, we aimed to provide a more focused comparison of the risks of the outcomes between the different AF subtype groups (AFADS vs. non-AF, KAF vs. non-AF, and AFADS vs. KAF), as these provide a clearer picture of the relative risks of the outcomes across the different groups. For these pairwise comparisons, we conducted separate Cox regression models with different reference groups, adjusting for the same set of covariates as in Tables 2-4.

The slight differences in the reported hazard ratios for AFADS and KAF versus non-AF between Tables 2-4 and Table 5 can be attributed to the different model specifications. We have updated the Results section accordingly: “Table 5 presents the adjusted hazard ratios for the pairwise comparisons of recurrent ischemic stroke, hemorrhagic stroke, and all-cause mortality risks between the AF subtype groups. Compared to patients without AF, both AFADS and KAF were associated with significantly higher risks of all outcomes. In the direct comparison between AFADS and KAF, the patients with KAF had lower risks of recurrent IS (HR: 0.91, 95% CI: 0.86–0.97, *p* < 0.01) and hemorrhagic stroke (HR: 0.88, 95% CI: 0.79–0.99, *p* < 0.01) and a higher risk of all-cause mortality (HR: 1.11, 95% CI: 1.07–1.16, *p* < 0.001).” (Please see Manuscript_Clean file P8L3-10)

We believe that this revised presentation of the results, along with the clarification of the AF subtype as an independent predictor in the Cox regression models, will improve the clarity and interpretability of our findings. Thank you for bringing this issue to our attention and allowing us to refine our manuscript.

**6) Since the mortality rate for KAF is particularly high, the risk of competing events strongly limits the results regarding the recurrence of Ischemic stroke and hemorragic stroke. I suggest that reporting “Compared with AFADS, the patients with KAF had lower risks of recurrent IS (HR: 0.91, 95% CI: 0.86–0.97, p < 0.01) and hemorrhagic stroke (HR: 0.88, 95% CI: 0.79–0.99, p < 0.01)” may be misleading. I also suggest to provide potential strategies for mitigating the impact of competing events on the study outcomes, thus strengthening the validity and reliability of the results of the study.**

**Reply:** Thank you for this insightful comment on the potential impact of competing risks on our study results, particularly regarding the comparison of recurrent ischemic stroke and hemorrhagic stroke risks between the AFADS and KAF groups.

To address this concern, we have now conducted additional analyses using the “Cox regression model” which accounts for the competing risk of death when estimating the hazard ratios for recurrent ischemic stroke and hemorrhagic stroke. This approach provides a more accurate assessment of the relative risks of these outcomes between the AF subtype groups in the presence of the competing event of death.

The results of the analysis are now presented in a new table (S2 Table). After accounting for the competing risk of death, the HR for recurrent ischemic stroke in KAF versus AFADS was 0.87 (95% CI: 0.82-0.92, p<0.01), indicating a significantly lower risk of recurrent ischemic stroke in the KAF group compared to the AFADS group. For hemorrhagic stroke, the HR for KAF versus AFADS was 0.91 (95% CI: 0.84-0.99, p<0.05), suggesting a slightly lower risk in the KAF group. In the Results section, we have added the following statement: "After accounting for the competing risk of death using the Cox regression model (S2 Table), the HR for recurrent ischemic stroke in KAF versus AFADS was 0.87 (95% CI: 0.82-0.92, p<0.01), and the HR for hemorrhagic stroke was 0.91 (95% CI: 0.84-0.99, p<0.05), indicating that patients with KAF have a lower risk of both recurrent ischemic stroke and hemorrhagic stroke compared to those with AFADS."

In the Discussion section, we have now acknowledged the potential impact of competing risks and the importance of using appropriate statistical methods to address this issue. We have added the following paragraph: “However, the results of the competing risk analysis (S2 Table) showed that that the higher mortality rate in the KAF group did not substantially bias the comparison of stroke risks between the AFADS and KAF groups. The reasons behind the elevated risk of recurrent strokes in patients with AFADS warrant further investigation to better understand the pathophysiology and guide targeted interventions.”

We believe that presenting the results of the competing risk analysis will provide a more balanced and accurate interpretation of our findings. Thank you for bringing this important methodological issue to our attention and giving us the opportunity to strengthen the validity of our study. Please let us know if you have any further questions or suggestions.

**7)** **The authors also reported that OAC therapy reduced the risk of all the outcomes of the study. However, they do not provide data regarding the population in which OAC therapy was studied. The results reported in the tables, suggest that they included in the cox regression analysis the whole population, also those with Non-AF, for which OAC therapy may not be indicated or beneficial. Please provide this data.**

**Reply:** Thank you for raising this important point about the population in which the effect of oral anticoagulant (OAC) therapy was studied. We apologize for not providing clear information on this in our original submission. You are correct that including patients without AF (non-AF group) in the analysis of OAC effectiveness may not be appropriate, as OAC therapy is not indicated for this population. To address this concern, we have now conducted additional analyses to assess the impact of OAC therapy on the outcomes specifically in the population with AF (i.e., AFADS and KAF groups combined). We have updated the Methods section to clarify this approach: "We also conducted multivariable Cox regression analyses in the subpopulation of patients with AF (AFDAS and KAF groups combined) to evaluate the effectiveness of OAC therapy in reducing the risks of recurrent ischemic stroke, hemorrhagic stroke, and all-cause mortality." (Please see Manuscript_Clean file P6L5-8)

In the Results section, we have added the following statement: "Cox regression model analysis of the effectiveness of OAC therapy in only patients with AF showed that OAC treatment was associated with significantly lower risks of recurrent ischemic stroke (HR: 0.20, 95% CI: 0.19-0.21, p<0.001), hemorrhagic stroke (HR: 0.20, 95% CI: 0.18-0.22, p<0.001), and all-cause mortality (HR: 0.26, 95% CI: 0.25-0.27, p<0.001) compared to no OAC therapy, after adjusting for potential confounders. (S3 Table) The benefits of OAC therapy were similar in both KAF and AFDAS subgroups (S4 Table and S5 Table)" (Please see Manuscript_Clean file P8L17-23)

In the Discussion section, we have now clarified the interpretation of our findings on OAC effectiveness: "Our study demonstrates the benefits of OAC therapy in reducing the risks of recurrent ischemic stroke, hemorrhagic stroke, and all-cause mortality in patients with AF, including both AFADS and KAF groups. These findings highlight the importance of prompt initiation and maintenance of OAC treatment in this high-risk population, in line with current guidelines." (Please see Manuscript_Clean file P12L7-11)

We believe that these revisions will provide a clearer and more accurate representation of the impact of OAC therapy on the outcomes in the relevant population. Thank you for bringing this issue to our attention and giving us the opportunity to improve the validity and interpretability of our findings. Please let us know if you have any further questions or suggestions.

**Minor
1) Discussion paragraph, page 8. “The incidences of AFADS (4.9%) and KAF (10.5%)…”. Maybe, it was meant “The prevalence”?**

**Reply:** Thanks. We have corrected it.

**2) I also suggest in the discussion in the paragraph regardin OAC therapy to refer to this relevant paper on the argument “ Romiti GF, Corica B, Proietti M, Mei DA, Frydenlund J, Bisson A, Boriani G, Olshansky B, Chan YH, Huisman MV, Chao TF, Lip GYH; GLORIA-AF Investigators. Patterns of oral anticoagulant use and outcomes in Asian patients with atrial fibrillation: a post-hoc analysis from the GLORIA-AF Registry. EClinicalMedicine. 2023 Aug 25;63:102039. doi: 0.1016/j.eclinm.2023.102039. PMID: 37753446”.**

**Reply:** Thank you for the comment. We added the findings form GLORIA-AF study (cited reference No. 38) in the paragraph discussing the rate of OAC use. (Please see P12L21-26) We specified why we only quoted two references (No. 7 and 19) in our original manuscript because these 2 references recruited similar patients (with ischemic stroke and AF) as ours.

**3) Also, it may be interesting to refer to some gender disparities regarding the risk of adverse events. Here I provide a relevant article on the topic “Bucci T, Shantsila A, Romiti GF, Teo WS, Park HW, Shimizu W, Mei DA, Tse HF, Proietti M, Chao TF, Lip GYH; Asia-Pacific Heart Rhythm Society Atrial Fibrillation Registry Investigators. Sex-related differences in presentation, treatment, and outcomes of Asian patients with atrial fibrillation: a report from the prospective APHRS-AF Registry. Sci Rep. 2023 Oct 26;13(1):18375. doi: 10.1038/s41598-023-45345-3. PMID: 37884587; PMCID: PMC10603128.”**

**Reply:** Thank you for the comment. We add a brief discussion about the relationship of gender and the risks of adverse events and our findings in the Discussion section (Please see Manuscript_Clean file P12L33-38 and P13L1-2). and cited some related references (No. 39-44).

**Reviewer #2**: Dear Authors, thank you for submitting your valuable study.
You have the unquestionable merit of having addressed a still unclear subject, the management of which is still too arbitrary and burdened by mortality and morbidity rates still too high.
However, I have some suggestions to offer you to refine the article and enrich the discussion section:

**Reply:** Thanks for your valuable suggestions that help us to polish our work.

**Q1: consideration should be given to the possibility that the results of the higher incidence of recurrent ischemic or hemorrhagic stroke in patients with AFDAS may be linked to the fact that atrial fibrillation in this cohort may recognize different etiopathogenic mechanisms (such as neurogenic atrial fibrillation). Similarly, cerebrovascular relapses may imply a different pathological pathway than atrial fibrillation in this cohort. The real challenge, in my opinion, will be to be able to distinguish how many patients with AFDAS recognize a cardioembolic genesis of their neurological events. This aspect should be underlined and deepened in the discussion;**

**Reply:** Thanks for raising the very insightful point. To address this point, we have now expanded the Discussion section (Please see Manuscript_Clean file P10L4-14) to highlight the potential role of different etiopathogenic mechanisms in the AFADS cohort and the potential mechanisms underlying the higher incidences of recurrent ischemic and hemorrhagic stroke in patients with AFADS:

“In addition, the underlying mechanisms of recurrent strokes in patients with AFDAS may be heterogeneous. Patients with neurogenic AF, who developed AF resulting from a stroke, may have non-cardioemoblic strokes. Similarly, some recurrent cerebrovascular events in patients with AFDAS may be related to non-cardioembolic pathways, such as atherosclerosis or small vessel disease, rather than AF itself. Distinguishing the proportion of AFADS patients with a true cardioembolic genesis of their neurological events remains a major challenge. Nonetheless, our findings suggest that AFADS patients represent a high-risk group that warrants close monitoring and aggressive secondary prevention measures, regardless of the precise etiology of their AF or stroke. Future studies incorporating these additional diagnostic modalities are needed to optimize risk stratification tools and therapeutic approaches.”

The paragraph provides a more balanced and informative discussion of the complex relationship between AF and stroke. We believe that acknowledging the potential different mechanisms and highlighting the challenges in distinguishing cardioembolic versus non-cardioembolic stroke in AFADS patients will provide a more nuanced interpretation of our findings. Thank you for raising this important point and giving us the opportunity to deepen our discussion of the complexities surrounding AFADS. Please let us know if you have any further questions or suggestions.

**Q2: another aspect to note is that only cases of atrial fibrillation diagnosed during the admission for the neurological event have been taken into account in the AFDAS group. As evidenced by many studies and as emerged with increasing strength thanks to the use of devices for the continuous monitoring of heart rhythm (such as implantable cardiac monitors or ECG-monitors for personal use), many patients with stroke have paroxysms of arrhythmia even at a distance from the index hospitalization. Therefore, many patients with AFDAS may have merged into the "non-AF" group. This should be highlighted within the limitations of the study and further discussed;**

**Reply:** Thank you for highlighting this important limitation of our study. We highlight the possible bias of underdiagnosed AFADS due to inadequate continuous cardiac monitoring in the paragraph of study limitation (Please see Manuscript_Clean file P13L13-23).

“The AFDAS group only included patients diagnosed with AF during the index hospitalization for stroke in this study. Patients who had paroxysmal AF or delayed onset AF after discharge may be classified as non-AF. Recent studies using continuous cardiac monitoring devices have shown that a significant proportion of patients with cryptogenic stroke may have undetected AF episodes in the weeks and months following the initial event. The limited use of long-term cardiac monitoring probably underestimated the true prevalence and impact of AF in this population. Future studies using prolonged cardiac monitoring techniques are needed to more accurately characterize the incidence and timing of AF in patients with stroke, and to evaluate the implications for risk stratification and management.”

This paragraph acknowledges the potential for misclassification of paroxysmal AF cases in our study and highlights the need for future research using more comprehensive AF detection methods. Please let me know if you would like me to modify this paragraph further.

**Q3: it would also be interesting to investigate the incidence of major adverse cardiovascular events during the follow-up period in the three patient groups. Would it be possible to add this analysis to the study?**

**Reply:** Thanks for this opinion. Based on the results of current study, it is of great importance to know the major causes of death and major adverse cardiovascular events in patients with IS and AF, in order to figure out what we can do to help more patients. We are currently working on these issues. The related data are complex and the major adverse events changes with time after the index stroke. These findings are difficult to summarize in a few sentences to be presented in the current manuscript. We would draft these finding in another manuscript.

Q4: The acronym AFADS does not sound right. I would suggest to modify it with AFDAS (Atrial Fibrillation Detected After Stroke), as widely reported in the literature. It also appears necessary, in conclusion, a minor revision of some typing and consecutio temporum errors of some sentences.

**Reply:** Thanks for the suggestions. We use AFDAS instead of AFADS in the revised manuscript, and check typing and typing and consecutio temporum errors with the help of commercial software and professional English editor.

Submission instruction: While revising your submission, please upload your figure files to the Preflight Analysis and Conversion Engine (PACE) digital diagnostic tool, <https://pacev2.apexcovantage.com/>. PACE helps ensure that figures meet PLOS requirements. To use PACE, you must first register as a user. Registration is free. Then, login and navigate to the UPLOAD tab, where you will find detailed instructions on how to use the tool. If you encounter any issues or have any questions when using PACE, please email PLOS at [figures@plos.org](mailto:figures@plos.org). Please note that Supporting Information files do not need this step.

**Q1:** We note that the grant information you provided in the ‘Funding Information’ and ‘Financial Disclosure’ sections do not match. When you resubmit, please ensure that you provide the correct grant numbers for the awards you received for your study in the ‘Funding Information’ section.

**Reply**: Thank you . We had revised the ‘Financial Disclosure’ and provided the correct grant numbers in the ‘Funding Information’ section.
